# Automated Design of Synthetic Gene Circuits in the Presence of Molecular Noise

**DOI:** 10.1021/acssynbio.3c00033

**Published:** 2023-10-09

**Authors:** Carlos Sequeiros, Carlos Vázquez, Julio R. Banga, Irene Otero-Muras

**Affiliations:** † Computational Biology Lab, MBG-CSIC, Spanish National Research Council, 36143 Pontevedra, Spain; ‡ Department of Mathematics and CITIC, Universidade da Coruña, 15071 A Coruña, Spain; § Computational Synthetic Biology Group, Institute for Integrative Systems Biology: I2SysBio (CSIC-UV), 46980 Valencia, Spain

**Keywords:** genetic design automation, molecular noise, stochastic dynamics, robust oscillator, biochemical adaptation, toggle switch

## Abstract

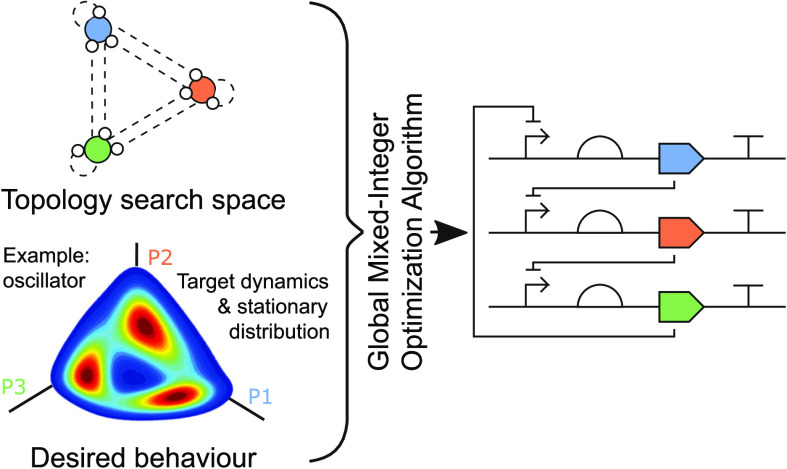

Microorganisms (mainly bacteria and yeast) are frequently used as hosts for genetic constructs in synthetic biology applications. Molecular noise might have a significant effect on the dynamics of gene regulation in microbial cells, mainly attributed to the low copy numbers of mRNA species involved. However, the inclusion of molecular noise in the automated design of biocircuits is not a common practice due to the computational burden linked to the chemical master equation describing the dynamics of stochastic gene regulatory circuits. Here, we address the automated design of synthetic gene circuits under the effect of molecular noise combining a mixed integer nonlinear global optimization method with a partial integro-differential equation model describing the evolution of stochastic gene regulatory systems that approximates very efficiently the chemical master equation. We demonstrate the performance of the proposed methodology through a number of examples of relevance in synthetic biology, including different bimodal stochastic gene switches, robust stochastic oscillators, and circuits capable of achieving biochemical adaptation under noise.

## Introduction

Automated genetic circuit design is a pivotal focus in synthetic biology, where the pursuit of increasingly sophisticated and well-conceived designs, coupled with rationalization, plays a critical role in accelerating the research cycle of design–build–test and learn.^1^

A milestone advance in biodesign automation is Cello, developed by the CIDAR Lab.^2^ Cello converts design specifications of combinational logic (input–output steady-state behavior) into DNA sequences, being the first automated design tool to have been experimentally calibrated and tested for predictability (in *Escherichia coli*). The implementation of Boolean logic circuits in different hosts and contexts is leading to important advances in the field.^3,4^

In the quest for a design framework allowing high levels of complexity in terms of nonlinear dynamics and more sophisticated cellular tasks, Otero-Muras et al.^5^ developed SYNBADm, combining modular ODE modeling and MINLP optimization. SYNBADm enables the *in silico* design of biocircuits of high complexity and multiple design objectives,^6^ exploiting MINLP solvers to optimize simultaneously acrosss parameter and topology spaces. It relies on models of ordinary differential equations; thus, it is suitable for deterministic regimes but cannot handle highly noisy cellular contexts, where stochasticity is relevant.

Here, we focus specifically on addressing the automated design of biocircuits in the presence of significant molecular noise. The function of a genetic circuit depends on both the reaction constants of the involved biomolecular processes and the topology of the regulatory interactions between genes. Therefore, similar to SYNBADm, we formulate the design as a mixed integer nonlinear programming optimization problem with real design variables (representing the parameters) and integer design variables (representing the topology) and optimize simultaneously through topology and parameter spaces.

From the computational side, the main difficulty to overcome is the computational burden associated with the simulation of inherently stochastic gene regulatory networks. The direct simulation of the chemical master equation describing the dynamics of these systems becomes intractable in realistic scenarios, and the existing approximations might be computationally expensive when dealing with optimization or real-time control problems in which a high number of simulations needs to be performed inside of an optimization algorithm. In order to simulate the dynamics of stochastic gene regulatory networks more efficiently, we exploit the method developed by Pajaro and co-authors.^7^ This method offers an accurate approximation of the chemical master equation using partial integro-differential equations (PIDEs). These equations can be effectively solved through a semi-Lagrangian method, as implemented in SELANSI by Pajaro and co-authors.^8^ Additionally, in this work, we parallelize the method on GPUs to achieve further acceleration.

In summary, the method presented in this paper formulates the automated design of biocircuits as an optimization problem wherein the cost function encodes the design objectives defined *a priori*, exploiting PIDE models^7^ to efficiently simulate the stochastic gene circuit dynamics and MINLP optimization solvers^9^ to optimize simultaneously through parameter and topology spaces.

We illustrate the performance of the proposed methodology through the design of a number of biocircuits with different target behaviors of interest for synthetic biology, including (1) circuits with prescribed target protein probability distributions or probability distributions with prescribed properties, such as specific relationships between the density of the modes or prescribed probabilities within specific domains, (2) circuits with adaptation capability in the presence of molecular noise, and (3) stochastic oscillators with optimal robustness against molecular noise.

## Methods

### Chemical Master Equation (CME) and the Stochastic Simulation Algorithm (SSA)

In the context of high molecular noise, a biocircuit is a stochastic gene regulatory network with dynamics described by the time evolution of a probability distribution. A discrete-state stochastic reaction network represents a reactive system^10^ with *N* species, each with a total number of *X*_*n*_ copies, the numbers of which evolve through *M* reactions, represented by *N*-dimensional vectors  that can fire in a differential time interval with some probability.

The probability of a discrete stochastic gene regulatory system being in some state **X** at a time *t* is denoted by *P*(**X**,*t*) and satisfies the following chemical master equation (CME), as introduced in Ge and Qian^11^
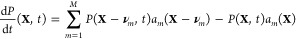
1where **X** = (*X*_1_,···*X*_*N*_) and *a*_*m*_ is the propensity of the *m* reaction, i.e., the probability of that biochemical reaction firing in the time interval [*t*, *t* + d*t*].

The probability distribution satisfying the CME can be exactly sampled, obtaining feasible temporal evolutions (realizations) of the number of molecules of each species using the stochastic simulation algorithm (SSA).^12^ The SSA is a Monte Carlo method that allows obtaining exact samples from the solution of the CME and mainly consists of randomly selecting the time of the next reaction by sampling an exponential distribution whose mean is the inverse of the sum of the propensities of the reactions and selecting the next reaction with a probability proportional to its propensity.

### Partial Integro-Differential Equation (PIDE) Model

As an alternative to SSA, and due to the intractability of the CME in realistic scenarios, we employ an approximation of the CME for gene regulatory networks in the form of a partial integro-differential equation (PIDE) developed by Pájaro et al.^7^
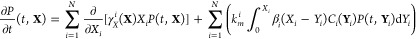
2
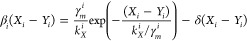
3where **X** is a bounded continuous approximation to the system’s state space, *k*_*m*,*X*_^*i*^ (γ_*m*,*X*_^*i*^) are respectively the production (degradation) rates of the mRNA and protein associated with gene *i* and δ(*X*_*i*_ – Y_*i*_) denotes the Dirac delta distribution. This PIDE model is numerically solved by means of a semi-Lagrangian scheme that has been implemented here in GPU in order to maximize its performance. Solving the PIDE we get the time series of the full probability density function (in contrast to other methods based on Monte Carlo sampling or on the distribution moments^13^).

In order to model the transcriptional interactions, we used Hill kinetics. Following the formalism used by Pájaro et al.,^7^ the probability of a gene being in the inactive state, due to the action of some protein is
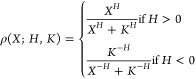
4where *X* represents the number of proteins, *H* is the integer Hill coefficient (quantifying the degree of cooperativity of the interaction), and *K* is a real positive coefficient, the geometrical mean of the reaction constants of each protein binding. If *H* > 0, the regulation is negative (repression), while if *H* < 0, the regulation is positive (activation). Note that for *H* ≠ 0, ρ(*X*; *H*,*K*) = 1 – ρ(*X*; −*H*,*K*). The transcriptional interactions as well as the parameters in eq 4 are represented by two matrices,  and , where the element *ij* represents respectively the integer and real coefficient of the regulation over the *i*-th gene by the protein transcribed by *j*-th gene. The total regulation (probability of activation) of a given gene *i* is given by the following multivariate function.^14^
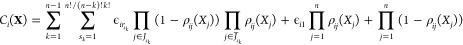
5The activation functions *C*_*i*_(**X**) can also be defined by the formula:
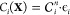
6where the vector ϵ_*i*_ represents all the leakage coefficients for all possible regulation states of gene *i* and  is calculated by recursively applying the formula.
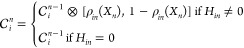
7
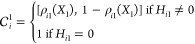
8The operator ⊗ represents the Kronecker product and ρ_*in*_(*X*_*n*_) = ρ(*X*_*n*_; *H*_*in*_,*K*_*in*_). Unlike the previous formulation defined in eq 6, the ϵ factor multiplying the term proportional to ∏_*j*=1_^*n*^(1 – ρ_*ij*_(*X*_*j*_)) (which represents the state of full activation) is not assumed to be 1. Unless stated otherwise, the initial condition for the simulations is a normal probability density with parameters **μ** = [10, ···10] and variance **σ** = [5, ···5]. It is important to remark that the initial condition does not affect the final stationary distribution.^15^

### Stochastic Biocircuit Design as an Optimization problem

The design of a biocircuit is formulated as an optimization problem with a cost function that depends on the target (i.e., the desired behavior). The design variables will also depend on the problem (commonly some of the parameters including degradation, leakage constants, etc. are fixed and not considered as degrees of freedom for the design). For the sake of generality, we consider here the case with all the parameters as design variables and formulate the general optimization-based design problem as
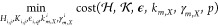
subject to eqs 2, 3, and 5, jointly with the following additional constraints
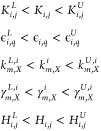
9where *H*_*i*,*j*_ represents the integer Hill coefficient of the regulation over the gene *i* by gene *j*, *K*_*i*,*j*_ represents the real positive coefficient of the same regulation, ϵ_*i*,*q*_ is the leakage coefficient of each possible regulation of gene *i*, *k*_*m*,*X*_^*i*^ are the production constants of the mRNA (m) and the protein (X) associated with gene *i*, and γ_*m*,*X*_^*i*^ are the corresponding degradation constants. This is a mixed integer nonlinear optimization programming problem. At each iteration of the optimization algorithm, the constraint imposed by the dynamics is computed by the PIDE model solver. We consider the following two alternative mixed integer encodings of the optimization problem:

- Encoding-I: the elements of *H* and *K* are multiplied by an integer design parameter in the set {−1, 0, 1} representing the sign of the regulation (in the case of *K*, its elements are multiplied by their absolute value).
- Encoding-II: we consider the elements of the *H* matrix to be positive integers and impose a lower bound for the *K* matrix close to zero. This allows the optimizer to eliminate a regulation between genes by choosing a low value of the *K* elements. Further, the leakage coefficients of each gene were added as design variables in order to choose the sign of each regulation.

Although encoding-II results in a larger mixed integer problem (more decision variables), it is less degenerate than encoding-I. In general, their performance will be problem dependent. However, we have tested both encodings in different scenarios, including all the case studies presented in the Results section, and as a general observation, encoding-I is preferable in the simpler two-dimensional (2D) problems where the design target is a specific probability density function, while encoding-II is recommended for more complex three-dimensional (3D) problems.

### Evaluation of the Fitness of the Design

When designing a synthetic gene regulatory circuit based on a target probability distribution, we try to minimize some measure of the divergence between the target probability distribution and the simulated one. We use two different measures, the integral of the squared difference between two distributions *f* and *g*
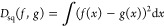
10and the Kullback–Leibler divergence^16^
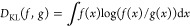
11Although more general divergence measures could be explored, Kullback–Leibler is the more popular one and is at the intersection of the classes of *f*-divergence and Bregman divergence measures,^17^ thus exhibiting the monotonicity property of the first class and the suitability for convex optimization problems of the second class. Also note that Kullback–Leibler divergence is a special case of α-divergence with α = −1. As illustrated in the forthcoming section of numerical examples, the use of Kullback–Leibler provides very satisfactory results in the solution of the different problems.

The method is flexible to design any target functionality, through the definition of different cost functions such that the desired behavior is achieved at the minimum cost. Next, we introduce cost functions corresponding to different functionalities of interest to be employed in the case studies tackled in this paper, including the design of toggle switches with different specifications (target dynamics, target stationary bimodal distribution, target modes), the design of circuits with capacity for biochemical adaptation, and the design of robust stochastic oscillators.

#### Bimodal Stochastic Gene Switches

When a stochastic switch is designed, one potentially relevant design criterion is the relative position of the modes (*X*_*i*_^*m*^) of the probability density function. For example, we might be interested in maximizing the distance between them, using as cost function
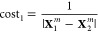
12In addition, we can include the proportion ρ between the probability density of the modes, as
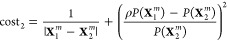
13or even the proportion σ between the probability density of a mode and the saddle point (**X**_sd_)
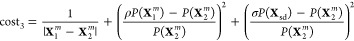
14

#### Detection of Extrema and Saddle Points

Among the most important parameters for approximately describing a 3D surface  i.e., a scalar function of a two dimension vector are its extrema and saddle points, and thus, detecting them is important for some design strategies (taking into account that surfaces enclose cell subpopulations). Due to the discrete character of the surfaces obtained by simulation, the extreme points will be located by comparing every point in the surface’s mesh to its neighbors closer than some distance *D*(*x⃗*, *y⃗*) = max_*i*_|*x*_*i*_ – *y*_*i*_| and checking if its value is higher than any of the neighbors.

Locating the saddle point of *g* is a more complex task, which we approach by constructing a helper function *f* that has a maximum in the same point where the problem function *g* has its saddle point. The helper function is constructed as follows
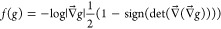
15where sign(*f*(*g*)) returns −1 if the argument is negative, 1 if it is positive, and 0 if it is zero. The factor −log|∇⃗*g*| guarantees maxima wherever the gradient vanishes, while the second one only is nonzero where the eigenvalues of the Hessian of *g* have opposite signs. We can also design a gene circuit based on the probability of certain subdomains of the state space, with the cost being
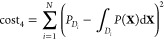
16where *P*_*D*_*i*__ is the target probability of the domain *D*_*i*_.

#### Biochemical Adaptation

In the case of the design of a stochastic gene circuit with adaptation capability, we try to optimize both the sensitivity (response to the stimulus) and the accuracy (capability to return to the previous state) of the system.^18^
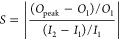
17
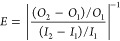
18where *O*_1_ is the protein level before the input, *O*_2_ is the level after the input effect has settled, and *O*_peak_ is the maximum response of the system (which may be a maximum or a minimum depending on the case), while *I*_1_ and *I*_2_ are the input levels before and after the induction, respectively. In order to optimize these two quantities, we use the same cost function as in Lormeau et al.^19^
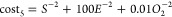
19Here, since the dynamics of the stochastic process are given by the evolution of a probability density function over time, the protein levels in eqs 17 and 18 are considered to be the expected values (i.e., weighted averages) of the distributions.

#### Stochastic Genetic Oscillator

The design of an oscillator requires simulating a potential trajectory of the system rather than a probability distribution in order to verify whether the level of the readout protein(s) oscillates over time. Previous approaches to identify stochastic oscillations include the measurement of mean periods and the expected values of the maximum and the minimum of the oscillation in order to compute the inverse of the average of the amplitude.^20^ Here, we use a very efficient way to identify stochastic oscillations based on the autocorrelation function.^21^
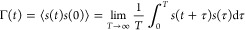
20After subtracting the mean and dividing the result by its global maximum, we get
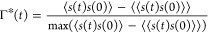
21This renormalization allows the comparison between oscillation robustness under the noise of different simulations, regardless of the amplitude or offset. The autocorrelation function of a deterministic oscillator is a harmonic oscillator. In contrast, in the presence of molecular noise (i.e., for a stochastic oscillator), the autocorrelation is a smooth but damped oscillation. Thus, as a design principle for oscillators, we maximize the second maximum of the autocorrelation function.^21^ We define this cost function to maximize as
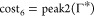
22

Importantly, the method not only finds efficiently stochastic oscillators but also finds oscillators that are optimal in terms of robustness against molecular noise. This trait is of particular relevance in bacterial and yeast synthetic oscillators, where often the quality of the oscillations is heavily affected by noise.

### Numerical Optimization Method

As previously stated, the optimization problem described in eq 9 involves real and integer decision variables, i.e., it is a mixed integer nonlinear programming (MINLP) problem. In addition, one of the constraints is given by a set of PIDEs describing the stochastic dynamics of the gene regulatory system, and therefore, we need to solve an MINLP problem with an embedded PIDE model. In order to solve efficiently this challenging nonconvex optimization problem, we use the hybrid optimization solver eSS by Egea et al.,^22^ which combines a global optimization metaheuristic (enhanced scatter search), in order to avoid the possibility of stagnation in a local minimum, with the local MINLP method MISQP (Exler and Schittkowski^23^) in order to refine the solution around the minima, as implemented in the MEIGO toolbox.^9^ This method outperforms other methods tested for similar tasks.^6^ In order to verify convergence of the method, multiple runs of each problem are executed. The results of the optimization-based design problems obtained by means of numerical global optimization techniques are reported in the next section. All computations were performed on a Dell precision T5500 workstation with two Intel Xeon E5645 CPUs at 2.40 GHz, a NVIDIA GeForce RTX 2080 Ti GPU, and 24 GB of DDR3 RAM memory, using MATLAB R2019b running under Windows 10. The source code used for the design is available at https://github.com/Carlos-Sequeiros/Automated-design-of-synthetic-gene-circuits-in-presence-of-molecular-noise.

## Results and Discussion

### Automated Design of Stochastic Gene Circuits with a Target Dynamic Behavior

As a first proof of concept of our method, we show the results obtained for the design of synthetic gene circuits (topologies and parameters) leading to a target dynamic behavior, i.e., with evolution of the cell population over time, in terms of protein expression levels, as close as possible to a given target time series.

**Figure 1:**
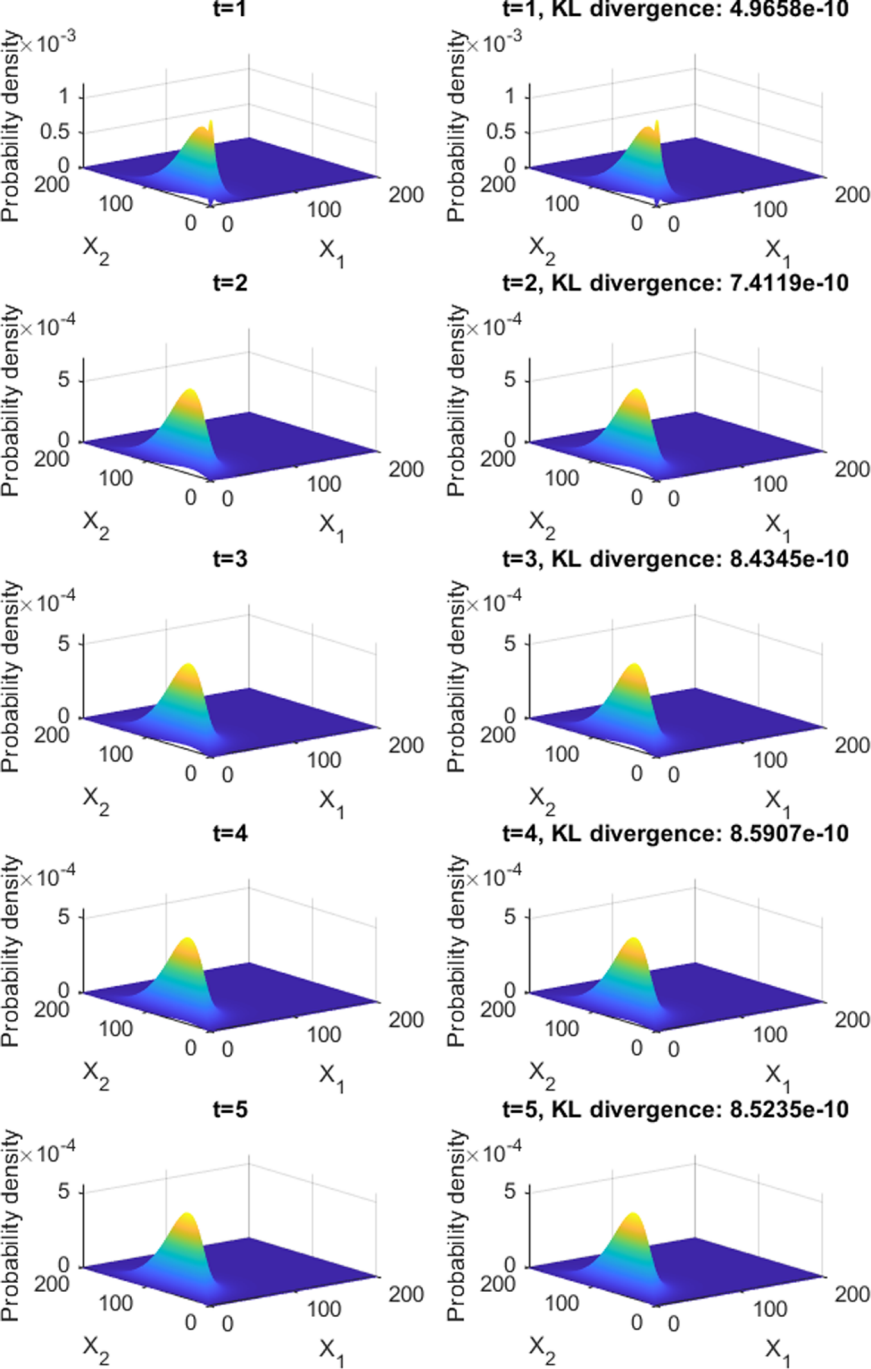
Comparison between the target temporal evolution of the protein probability density function (left) and the one obtained by optimization-based design (right). mRNA production (m), protein production (X), and degradation rate constants of mRNA and proteins being *k*_*m*_^1^ = 10, *k*_*X*_^1^ = 250, γ_*m*_^1^ = 25, γ_*X*_^1^ = 1, *k*_*m*_^2^ = 20, *k*_*X*_^2^ = 500, γ_*m*_^2^ = 25, γ_*X*_^2^ = 1, constant leakage ϵ = 0.15. Only 5 of the 10 snapshots are shown due to space limitation.

As target dynamics, we use the temporal evolution of the protein probability density function depicted in Figure 1. The dynamics are defined here by 10 snapshots at different times, obtained by simulation with SELANSI starting from a gene regulatory circuit with fixed production, degradation, and leakage constants. Note that the level of discretization used to define the target dynamics might affect the number of compatible designs (with equivalent performance).

We start from the superstructure of all the possible regulation topologies with 2 genes (including all the potential mutual and self-inhibition and activation interactions) and optimize across the parameter and topology spaces in order to find the circuit with the desired behavior.

We solve the optimization problem (9), with design variables being all the elements of matrices *H* and *K* (i.e., 4 integer and 4 real variables) with lower and upper bounds given by 1 ≤ *H*_*ij*_ ≤ 3 and 10 ≤ *K*_*ij*_ ≤ 200, as well as their sign −1 ≤ *Sg*_*ij*_ ≤ 1. The bounds on *K*_*ij*_ have been chosen to match the computational domain’s borders, large enough to capture any biologically relevant behavior. In the case of *H*_*ij*_, the range [1, 3] is enough to get into account all relevant behaviors. We use as cost function a measure of the divergence between the target probability distribution over time and the simulated one. We solved the optimization problem for both square distances (eq 10) and Kullback–Leibler distances (eq 11). Note that the cost for a given design is the sum of these metrics between distributions for each time point. Each evaluation of the cost function takes approximately 3 s in both cases. The simulations are performed with a spatial mesh *X*_1,2_ ∈ [0, 200]; Δ*X*_1,2_ = 0.5 and temporal mesh *t* ∈ [0, 5]; Δ*t* = 5 × 10^–3^. In order to guarantee the accuracy of the simulations, the bounds of the spatial mesh are chosen such that all states with a significant probability lie within the computational domain. The activation functions *c*_*i*_(**X**) are defined in the same computational domain, and the upper bounds on *K*_*ij*_ are chosen so that the whole range of input functions *c*_*i*_(**X**) is covered.

The optimization process takes an average of 3228 evaluations of the cost function for the squared difference and 3112 evaluations for the Kullback–Leibler divergence. Both methods led to good designs. In both cases, the dynamics obtained are practicably indistinguishable from the original target, see Figure 1. Remarkably, Kullback–Leibler divergence recovers almost exactly the parameters that have been used to define the target distribution: *K*_1,1_ = 45.0057, *K*_1,2_ = 45.0130, *K*_2,1_ = 70.0018, *K*_2,2_ = 69.9997, *H*_1,1_ = 3, *H*_1,2_ = 2, *H*_2,1_ = 3, *H*_2,2_ = 2, *Sg*_1,1_ = −1, *Sg*_1,1_ = 1, *Sg*_1,1_ = −1, *Sg*_1,1_ = 1.

### Automated Design of Stochastic Genetic Toggle Switches

#### Toggle Switch with a Target Dynamic Behavior and Stationary Bimodal Distribution

In a stochastic genetic toggle switch, the stationary distribution is bimodal. Next, we illustrate how to design a stochastic toggle switch with target dynamics and stationary bimodal distribution.

**Figure 2:**
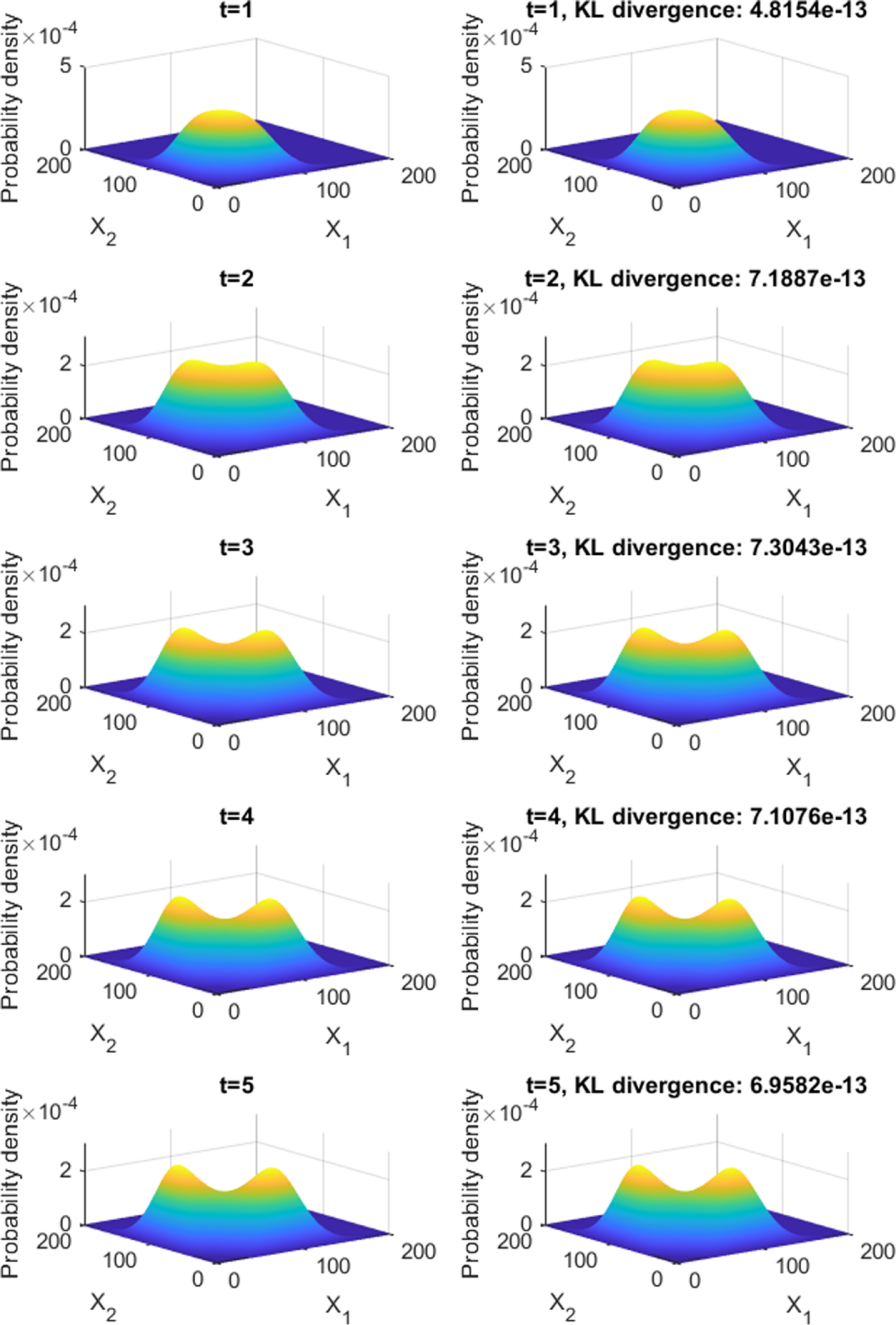
Comparison between the target temporal evolution of the bimodal protein probability density function (A) and the one obtained by optimization-based design (B). mRNA production (m), protein production (X), and degradation rate constants of mRNA and proteins being *k*_*m*_^1,2^ = 10, *k*_*X*_^1,2^ = 200, γ_*m*_^1,2^ = 25, γ_*X*_^1,2^ = 1, constant leakage ϵ = 0.1. Only 5 of the 10 snapshots are shown due to space limitation.

The target behavior, illustrated in Figure 2 A, is generated by simulation with SELANSI with fixed parameters.
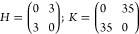
23We want to find among all the possible 2-gene regulatory configurations a gene circuit with the target dynamic and stationary toggle switch behavior. We solve the optimization problem (9), with design variables being all the elements of matrices *H* and *K* (i.e., 4 integer and 4 real variables) with lower and upper bounds given by 1 ≤ *H*_*ij*_ ≤ 3 and 10 ≤ *K*_*ij*_ ≤ 200 as well as their sign, – 1 ≤ *Sg*_*ij*_ ≤ 1. The employed criteria to choose the parameter bounds are the same as in the previous case. We use as a cost function a measure of the divergence between target and simulated probability distribution, summed over time. We have solved the design problem using square distances (eq 10) and Kullback–Leibler (eq 11). As design parameters, we use 1 ≤ *H*_*ij*_ ≤ 3 and 10 ≤ *K*_*ij*_ ≤ 200 and −1 ≤ *Sg*_*ij*_ ≤ 1.

Each evaluation of the cost function takes approximately 3 s for both squared distances and Kullback–Leibler. The optimization process requires an average of approximately 2500 evaluations of the cost function for both the squared distance and Kullback–Leibler divergence. The simulations are performed with a spatial mesh *X*_1,2_ ∈ [0, 200]; Δ*X*_1,2_ = 0.5 and a temporal mesh *t* ∈ [0, 5]; Δ*t* = 5 × 10^–3^, similarly as in the previous case.

In order to compare the efficiency of SELANSI with respect to SSA, achieving the same solution with SSA requires at least 10^6^ Monte Carlo simulations, ideally 10^7^ to get histograms clearly converging to the underlying probability distributions, which take around 6 and 37 s per cost function evaluation, respectively. Therefore, this makes around 4 and 25, 7 h, respectively, instead of the 2 h employed by SELANSI.

The obtained parameters are *K*_1,1_ = 13.4262, *K*_1,2_ = 35.0000, *K*_2,1_ = 35.0000, *K*_2,2_ = 51.6670, *H*_1,1_ = 1, *H*_1,2_ = 3, *H*_2,1_ = 3, *H*_2,2_ = 1, *Sg*_1,1_ = 0, *Sg*_1,1_ = 1, *Sg*_1,1_ = 1, and *Sg*_1,1_ = 0, and the distributions obtained are therefore identical (see Figure 2).

#### Toggle Switch with Target Modes and Saddle Points at the Stationary Distribution

With this design criterion, our aim is to maximize the distance between modes of a bimodal stationary protein probability distribution while also enforcing specific relationships between the probability density values of the modes and the saddle point. The design parameters employed for this problem are the elements of Hill matrix 1 ≤ *H*_*ij*_ ≤ 3 and 10 ≤ *K*_*ij*_ ≤ 1000 as well as their sign −1 ≤ *Sg*_*ij*_ ≤ 1, while setting the reaction constants to *k*_*m*_^1,2^ = 10, *k*_*X*_^1,2^ = 200, γ_*m*_^1,2^ = 25, and γ_*X*_^1,2^ = 1. In this case, we have chosen the bounds of the parameters large enough to capture any biologically relevant behavior. Our design criteria are, aside maximizing the distance between modes, that one mode’s probability density must be 3 times higher than the other (i.e., ρ = 3) and 10 times higher than that of the saddle point (i.e., σ = 10). We use the cost function defined in eq 14. The simulations are performed with a spatial mesh *X*_1,2_ ∈ [0, 200]; Δ*X*_1,2_ = 0.5 and temporal mesh *t* ∈ [0, 5]; Δ*t* = 5 × 10^–3^.

**Figure 3:**
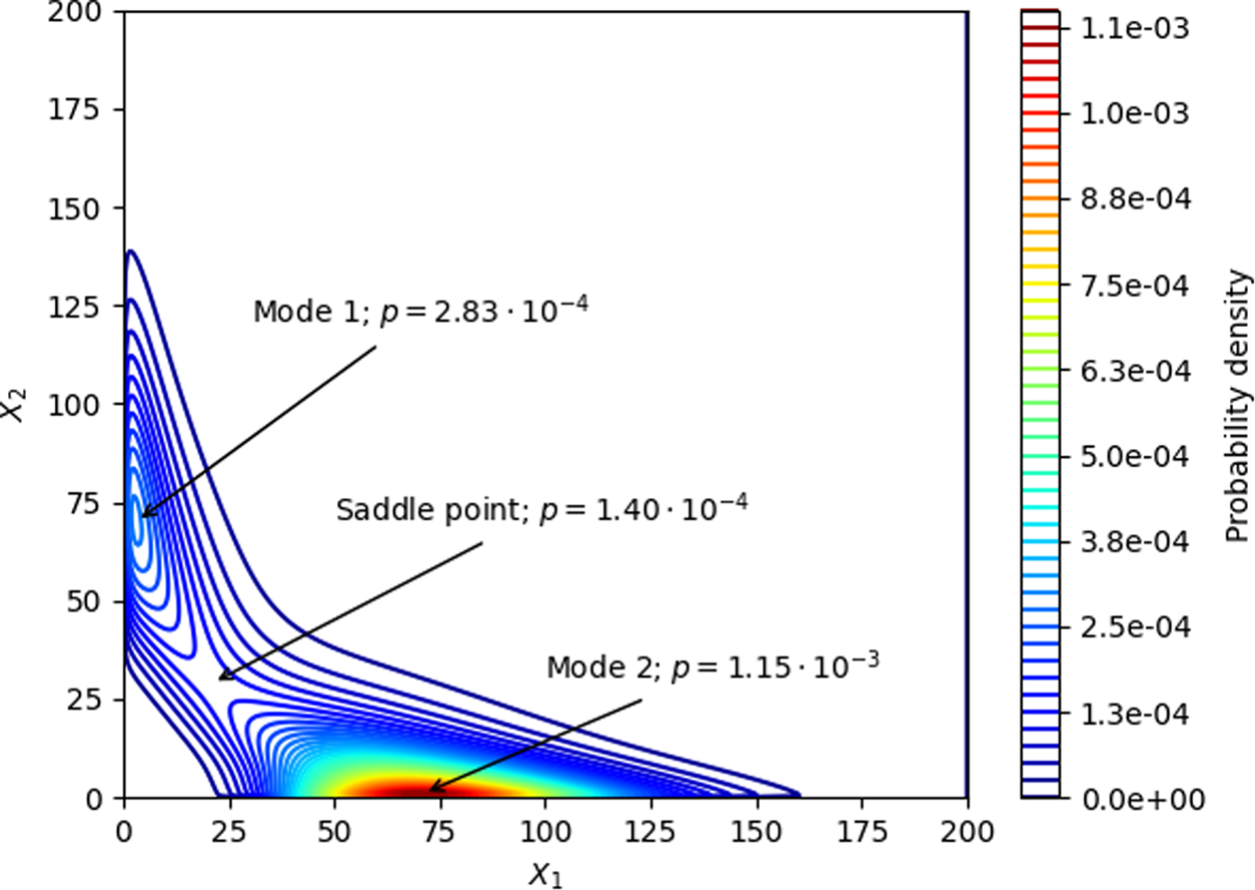
Stationary protein probability density function of the network designed maximizing the intermodal distance and prescribing relationships between the modes’ and chair point’s probability densities.

**Figure 4:**
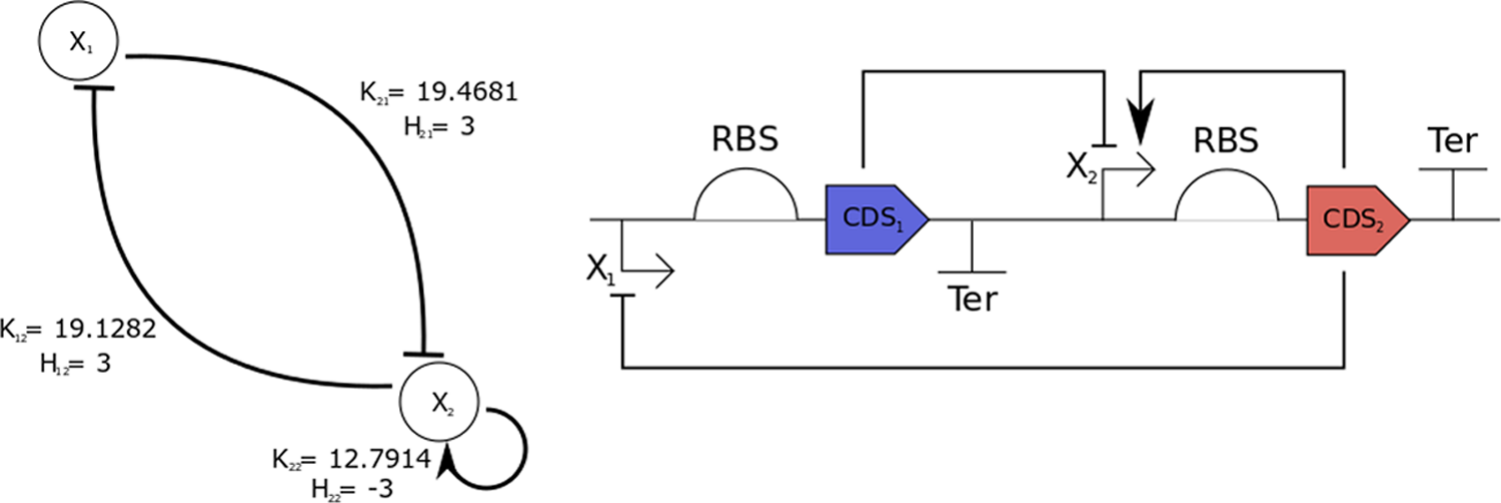
Topology of the toggle switch design based on mode and saddle point probability relationships and intermodal distance maximization. Interestingly, not all possible regulations are present.

As can be seen in Figure 3, the stationary protein probability density function has two modes, each one almost at the border of the computational domain, and the proportions between the modes’ and saddle point’s probability densities are close to that prescribed by design. The designed gene circuit is shown in Figure 4. Each evaluation of the cost function takes approximately 3.21 s in wall-clock time and the optimization process consumes a total of 3155 evaluations, lasting for 2 h and 49 min, although convergence can be assumed at 1533 evaluations. The obtained parameters are *K*_1,1_ = 10.0000, *K*_1,2_ = 19.1282, *K*_2,1_ = 19.4681, *K*_2,2_ = 12.7914, *H*_1,1_ = 1, *H*_1,2_ = 3, *H*_2,1_ = 3, *H*_2,2_ = 3, *Sg*_1,1_ = 0, *Sg*_1,1_ = 1, *Sg*_1,1_ = 1, and *Sg*_1,1_ = −1.

#### Toggle Switch with a Given Domain Probability as Target

Another potential objective with significant practical implications in the design of a stochastic toggle switch is the precise configuration of the probability domains of the stationary distribution. Using our method, we can determine the gene circuit that generates a stationary probability distribution, aligning the probabilities of the system being in specific domains as closely as possible with the target domains specified by the user.

**Figure 5:**
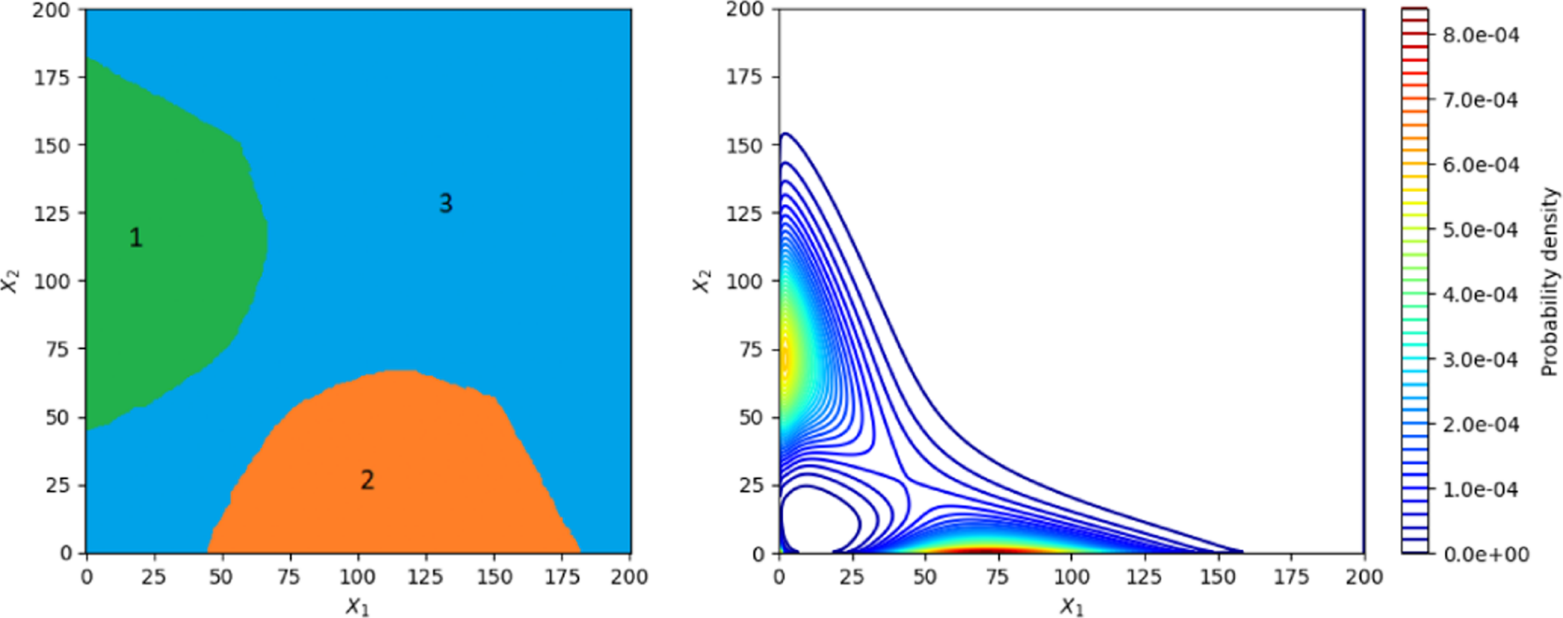
Left: the different domains in which the computational domain is divided. Right: stationary probability density function of the network designed by adjusting the total probability of the subdomains. The probability of the system being in domain 1 is 0.4925, 0.2903 in domain 2, and 0.2115 in domain 3.

We assign specific probabilities to subdomains (see Figure 5A) within some ranges of interest and use them as design criteria for the steady-state probability density of the gene circuit. As design variables, we employ all of the elements in matrix *H* and *K* multiplied by a sign (in the case of *K* its absolute value) while fixing reaction constants to *k*_*m*_^1,2^ = 10, *k*_*X*_^1,2^ = 200, γ_*m*_^1,2^ = 25, γ_*X*_^1,2^ = 1 and the leakage constants to ϵ = 0.1. The simulations are performed with a spatial mesh *X*_1,2_ ∈ [0, 200]; Δ*X*_1,2_ = 0.5 and temporal mesh *t* ∈ [0, 5]; Δ*t* = 5 × 10^–3^. We assign as target probabilities 0.5 to domain 1, 0.3 to domain 2, and 0.2 to domain 3. We use the cost function defined in eq 16. The bounds of the search domain are 1 ≤ *H*_*ij*_ ≤ 3, 10 ≤ *K*_*ij*_ ≤ 1000, and −1 ≤ *Sg*_*ij*_ ≤ 1, the same as in the previous case, applying the same criteria.

**Figure 6:**
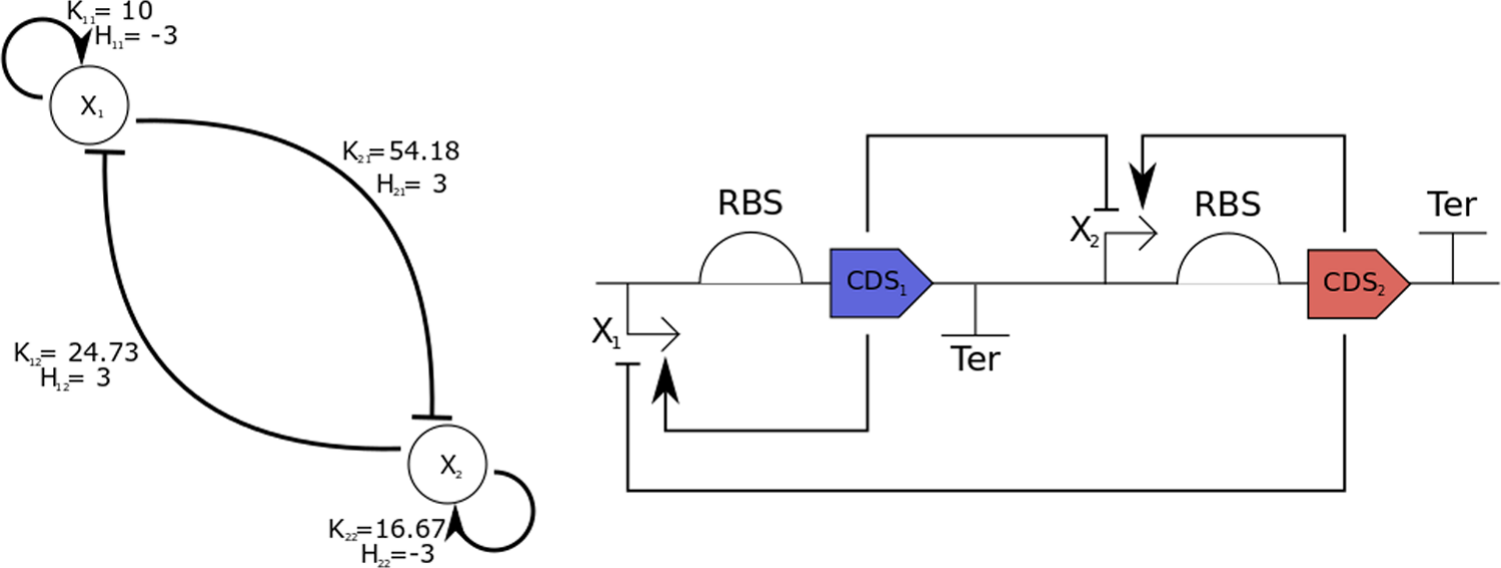
Synthetic gene regulatory network with optimal performance with respect to the prespecified target domain probabilities.

The stationary probability density function of the gene circuit found by the optimization-based design method is shown in Figure 5B. The synthetic gene regulatory circuit obtained is shown in Figure 6.

The synthetic circuit obtained shows a stationary protein probability density function with three modes, and the probability of the system’s state being inside each domain is very close to the predefined target. Each evaluation of the cost function takes about 2.4 s, and the optimization converged after approximately 900 evaluations (35 min in wall-clock time). The obtained parameters are *K*_1,1_ = 10.0001, *K*_1,2_ = 24.7301, *K*_2,1_ = 54.1827, *K*_2,2_ = 16.6711, *H*_1,1_ = 3, *H*_1,2_ = 3, *H*_2,1_ = 3, *H*_2,2_ = 3, *Sg*_1,1_ = −1, *Sg*_1,1_ = 1, *Sg*_1,1_ = 1, *Sg*_1,1_ = −1.

### Design of Gene Circuits with Adaptation Capacity

The capacity of cells for adaptation is an important capability extensively explored in the deterministic regime (in the absence of molecular noise).^18,19,24^ Here, we focus on designing circuits that enable cells to adapt to external perturbations, in the presence of molecular noise. This scenario is often more realistic in cases involving bacteria and yeast. To evaluate the capacity of adaptation, which includes sensitivity and precision as defined by Ma et al.,^18^ we have to take into account the stationary regime before the input, the peak response, and the stationary regime after the input. To this aim, we compare the expected levels of a readout protein. We use the cost function defined in eq 19.

We start from 3-gene regulatory topology in which the input gene is activated by an external inducer. The decision variables in this case are the elements in rows 2 and 3 of matrices *H* and *K*, as well as the leakage coefficients (including those corresponding to the full activation) of noninput genes, while the reaction parameters for all three genes are set to *k*_*m*_ = 8, *k*_*X*_ = 400, γ_*m*_ = 25, and γ_*X*_ = 1. The bounds of the search space are 0.01 ≤ *K*_*ij*_ ≤ 1000, 1 ≤ *H*_*ij*_ ≤ 4, and 0.1 ≤ ϵ_*ij*_ ≤ 1. The bounds of *K*_*ij*_ respond to the requirements of the encoding in this problem: those of ϵ_*ij*_ are a consequence of the meaning of the parameter (the ratio between the states of no activation and full activation), while those of *H*_*ij*_ reflect the need of including enough possible scenarios. The simulations are performed using a spatial mesh *X*_1,2,3_ ∈ [0, 350]; Δ*X*_1,2,3_ = 2.7344; and a temporal mesh *t* ∈ [0, 60], Δ*t* = 5 × 10^–3^.

The optimization algorithm performed 4908 evaluations of the cost function defined in eq 18. The decision vector obtained is *K*_21_ = 42.0397, *K*_22_ = 0.01, *K*_23_ = 0.01, *K*_31_ = 64.3775, *K*_32_ = 35.5506, *K*_33_ = 0.01, ϵ_21_ = 0.1, ϵ_22_ = 0.75787, ϵ_23_ = 1, ϵ_24_ = 1, ϵ_25_ = 1, ϵ_26_ = 0.10003, ϵ_27_ = 0.11217, ϵ_28_ = 0.4612, ϵ_31_ = 1, ϵ_32_ = 0.99289, ϵ_33_ = 0.1, ϵ_34_ = 0.99798, ϵ_35_ = 0.1, ϵ_36_ = 0.1, ϵ_37_ = 0.1, ϵ_38_ = 0.1286, *H*_21_ = 4, *H*_22_ = 1, *H*_23_ = 4, *H*_31_ = 4, *H*_32_ = 4, *H*_33_ = 2.

**Figure 7:**
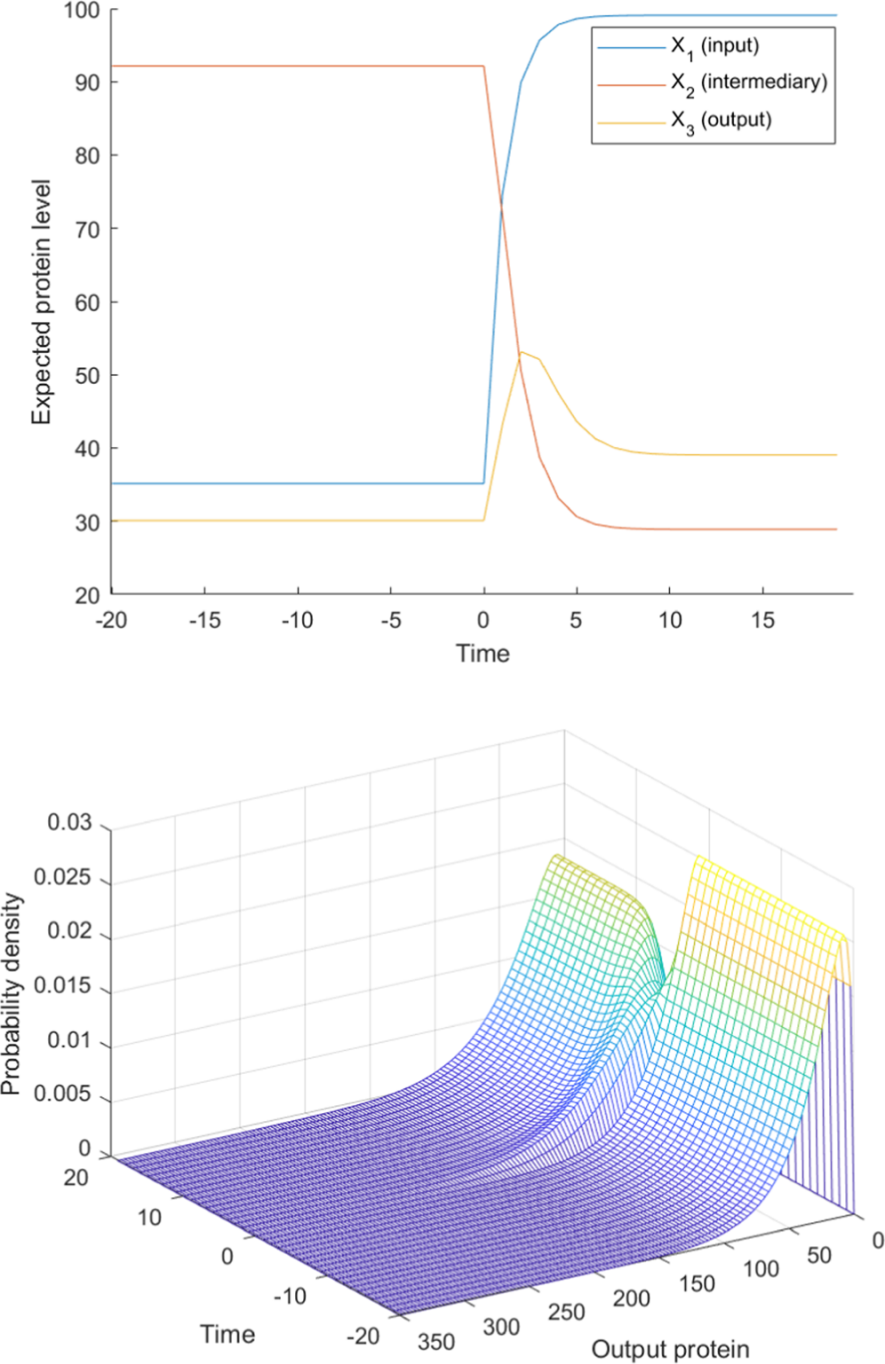
Top: expected (average) protein levels as a function of time. The average input protein level increases approximately by 200% upon induction. Bottom: time evolution of the output protein’s probability density. In both graphs, induction occurs at *t* = 0.

The activation functions of the second gene take the following form:
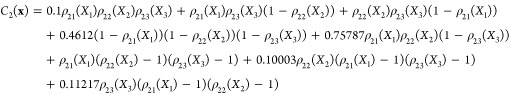
24Taking into account that every element in *H* is positive (thus the regulation is repressive) and the fact that, for low values of *K*, ρ(X_*i*_; *H*_*ij*_,*K*_*ij*_) ≃ 1, by substituting this result into eq 24 for ρ_22_(*X*_2_) and ρ_23_(*X*_3_) (as *K*_22_ = 0.01 and *K*_23_ = 0.01) resulting in
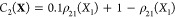
25which is the repression function for a single gene. Proceeding in a similar way with the activation function of the output gene, we obtain
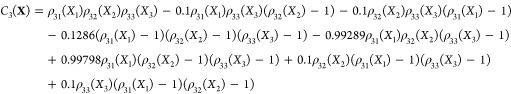
26Substituting ρ_33_(*X*_2_) in eq 26 by 1 as *K*_33_ = 0.01, we get
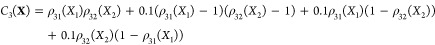
27This activation function is that of an activation by genes 1 (input) and 2 (intermediary). These activation functions correspond to an incoherent feed forward loop 4 (IFFL4), which is one of the known three-gene gene regulatory circuits capable of adaptation in the deterministic regime. This result indicates that IFFL4 is the best topology for adaptation in the stochastic regime (Figure 7).

### Design of Genetic Oscillators with Optimal Robustness against Molecular Noise

Since the first synthetic oscillator was implemented,^25^ important efforts and achievements have been made toward the design of oscillators, which are robust against molecular noise.^26^ Here, we address the problem of automated design of stochastic oscillators that are optimal with respect to their robustness against noise. As previously stated, to this aim, we maximize the second maximum of the autocorrelation function, using the cost function defined in eq 22. In this case, we fix the topology of the network to be consistent with the repressilator by Elowitz and Leibler.^25^ As design variables, we choose , , *k*_*m*_, *k*_*X*_, and γ_*m*_. The index in the reaction constants is omitted as it is the same for all genes, γ_*X*_ is fixed to 1, and all leakage coefficients ϵ are equal to 0.15.

For this case study, we set the parameter bounds to 3 ≤ *H* ≤ 7, 10 ≤ *K* ≤ 200, 3 ≤ *k*_*m*_ ≤ 120, 100 ≤ *k*_*X*_ ≤ 1000, and 4 ≤ γ_*m*_ ≤ 50. We have chosen the bounds for this problem based on the typical values of the parameters of previous simulations. The optimization algorithm converged after approximately 1000 cost function evaluations (about 5.5 h of wall-clock time). We obtain the decision parameters: *H* = 7, *K* = 199.8989, *k*_*m*_ = 113.4372, *k*_*X*_ = 100, and γ_*m*_ = 17.6822.

**Figure 8:**
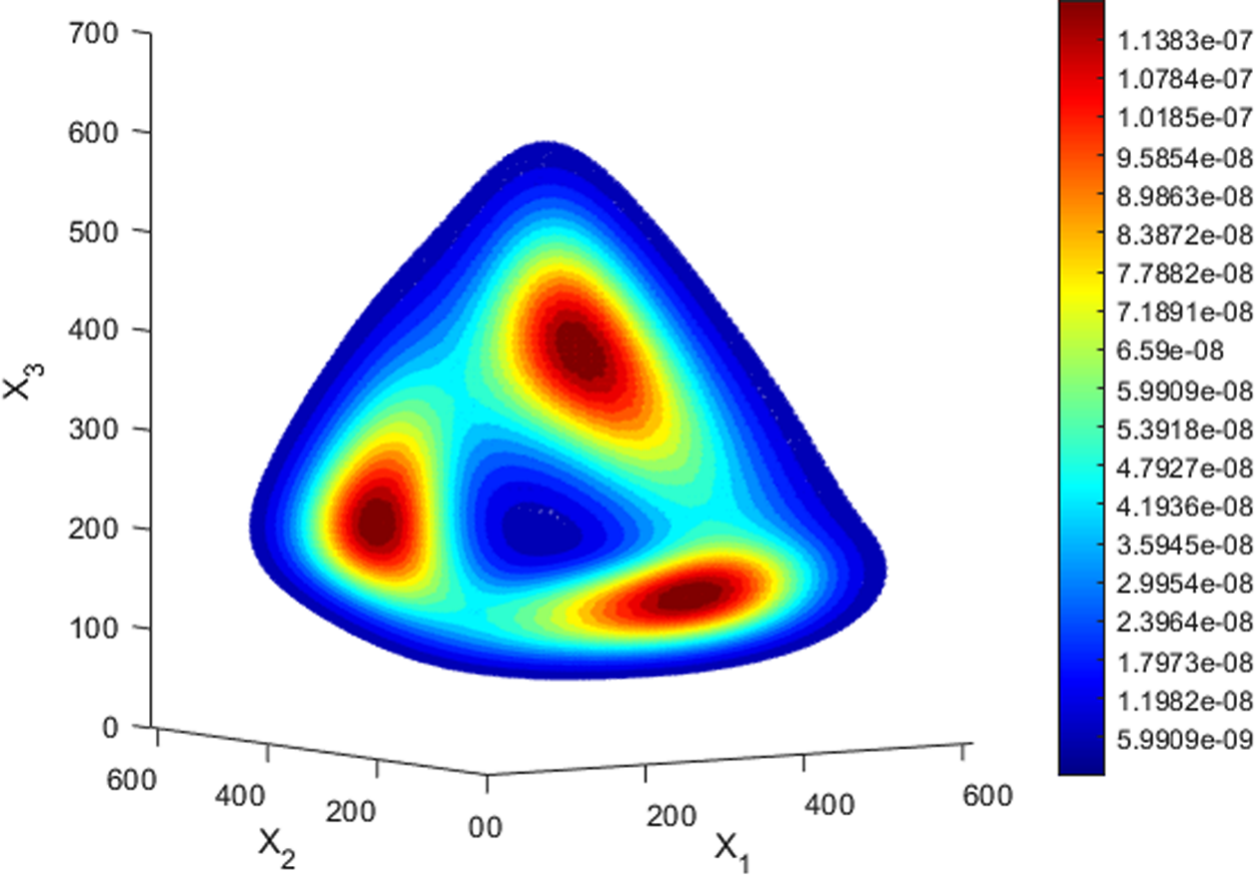
Isosurfaces of the stationary probability density function of the number of proteins in the designed repressilator.

The target system oscillates indefinitely, despite its probability density distribution reaching a stationary state. This phenomenon is caused by the phase degeneracy induced by noise, which eventually disrupts the coherence among different realizations, rendering all three maxima of the distribution equally probable, regardless of the initial conditions (Figure 8).

## Conclusions

During the recent years, many efforts have been made in developing methods and tools *in silico* to assist the design–build–test–learn process in synthetic biology.^27^ In spite of the advances on the modeling and design of biocircuits, including mechanistic model-based^27^ and machine learning-based methods,^28,29^ there are still significant challenges to overcome in effectively addressing inherent uncertainty, context dependency, and stochasticity of gene regulatory processes.

In this work, we develop a methodology for automated design of gene circuits in the presence of molecular noise. This problem has been addressed before for example by Woods et al.^20^ and Tanevski et al.^30^ The main distinctive features of the method presented here with respect to previous studies on automated design of stochastic biocircuits are

- the use of mixed integer nonlinear programming solvers, which enables one to optimize simultaneously across parameter and topology spaces, thus avoiding exhaustive exploration.
- the method for simulation of stochastic gene regulatory networks, based on partial integro-differential equations, provides an efficient computation of the probability distribution of the system over time.

To the best of the authors’ knowledge, this is the first tool for automated design combining stochastic simulation based on PIDE models with MINLP. Other optimization-based methods comparing probability distributions for stochastic model fitting^31^ use Monte Carlo methods to generate the distributions.

PIDE models allow obtaining the time series of probability distributions more efficiently than SSA. Therefore, we use PIDE models unless single realizations are preferred for some specific reason. Also, it is important to note that using the full distributions is highly recommended when the expected distributions are not normal, as it is generally the case in the simulation of gene regulatory networks^32^ (where we might find complex nonlinear behaviors like bimodality, which is not captured if we consider only the moments of the distribution^13^).

The combination of eSS with MISQP has been previously tested for computational efficiency and scalability standing out among other MINLP optimization alternatives.^33^ Here, we have also presented two different encodings for these problems, providing specific recommendations regarding their use.

As a proof of concept of our method, we considered the design of two-gene and three-gene circuits with target behaviors or functionalities of relevance for real-world synbio applications: switches for cell decision making processes at the level of bacterial and/or yeast populations, genetic oscillators in the presence of noise, and gene circuits endowing the host cell with the capacity of adaptation to perturbations.

In this way, the tool is also very valuable in order to elucidate design principles of gene regulatory circuits in the presence of molecular noise. Through our results, we have obtained, for example, switches with traits that are of particular relevance when designing cell decision making processes under molecular noise, including gene circuits for which the cell population stays in some subset of states for a specified fraction of the time or has a defined ratio between the probabilities of the modes. We have proved that a known topology for adaptation in the deterministic regime is also capable for adaptation in the presence of noise. Also, we show how to automatically design oscillators that are optimal in terms of their robustness against molecular noise. This result has very important implications not only toward the implementation of more robust oscillators in practice but also toward the elucidation of design principles underlying this essential trait for many biological oscillators.
